# Rapamycin Protects Skin Fibroblasts From UVA-Induced Photoaging by Inhibition of p53 and Phosphorylated HSP27

**DOI:** 10.3389/fcell.2021.633331

**Published:** 2021-02-05

**Authors:** Gen-Long Bai, Ping Wang, Xin Huang, Zi-Yue Wang, Di Cao, Chuan Liu, Yi-Yi Liu, Ruo-Lin Li, Ai-Jun Chen

**Affiliations:** ^1^Department of Dermatology, The First Affiliated Hospital of Chongqing Medical University, Yuzhong, China; ^2^Prescriptions Department, College of Traditional Chinese Medicine, Chongqing Medical University, Yuzhong, China

**Keywords:** rapamycin, UVA, photoaging, human dermal fibroblasts, Hsp27, autophagy

## Abstract

Skin aging caused by UV radiation is called photoaging is characterized by skin roughness and dryness accompanied by a significant reduction of dermal collagen. Rapamycin is a macrolide immunosuppressant which has been shown to exhibit “anti-aging” effects in cells and organisms, however, its roles in the skin photoaging remains unclear. Here, we investigate the role of rapamycin and HSP27, which we have previously identified as an inhibitor of UV-induced apoptosis and senescence in HaCat cells, in a UVA-induced photoaging model of primary human dermal fibroblasts (HDFs). Results from senescence-associated beta-galactosidase (SA-β-gal) staining revealed that rapamycin significantly reduced senescence in UVA-treated HDFs. In addition, treatment with rapamycin significantly increased cell autophagy levels, decreased the expression of p53 and phosphorylated HSP27, and reduced genotoxic and oxidative cellular stress levels in UVA-induced HDFs. Knockdown of HSP27 resulted in a significant increase of MMP-1 and MMP-3 as well as a decrease in type I collagen expression. Rapamycin mitigated these effects by activation of the classical TGF-β/Smad signaling pathway and increasing the transcriptional activity of MAPK/AP-1. Taken together, these results suggest that rapamycin may potentially serve as a preventive and therapeutic agent for UVA-induced photoaging of the skin.

## Introduction

Photoaging is the result of premature aging of the skin caused by the destructive effects of ultraviolet (UV) radiation. Distinctive clinical features of photoaging include a coarse, dry skin texture, pigmentation disorders, increased wrinkles, and telangiectasia (Young et al., 2017). Pathohistologically, photoaging is characterized by a significant reduction in dermal collagen and the accumulation of abnormal elastin fibers (Han et al., 2014). While the exact pathomechanisms remain unclear, high expression levels and increased activity of matrix metalloproteinases (MMP)-1, 3, and 9 have long been thought to play important roles in the process of photoaging (Nakyai et al., 2017; Seo et al., 2018).

In addition to its role as a molecular chaperone, our previous work revealed a role for HSP27, a member of the small heat shock protein family, in the regulation of apoptosis during skin photoaging. We found that HSP27 can mediate apoptosis in HaCat cells through the mitochondrial caspase pathway by activation of the Akt-dependent phosphorylation pathway and inhibition of the p53/Bax/Bcl-2-dependent mitochondrial apoptosis pathway in order to regulate the subcellular localization of p21 and thereby exerting photoprotective effects (Liu et al., 2019a,b). However, the role of HSP27 in dermal tissue photoaging, especially in UVA-irradiated human dermal fibroblast (HDF) photoaging remains unclear.

Autophagy is an evolutionarily conserved cellular process that is thought to be an essential cell survival mechanism via the degradation of unnecessary or dysfunctional organelles and proteins (Yonekawa and Thorburn, 2013). Decreased or defective autophagy has been linked to a variety of pathological processes, including neurodegeneration, infection, cancer, and aging (Mizushima and Komatsu, 2011). It has been demonstrated that the activation of autophagy can exert anti-apoptotic effects by facilitating UVA-induced clearance of oxidized phospholipid and protein aggregates from keratinocytes (Zhao et al., 2013). More recently, it has been shown that UVA-induced photoaging in fibroblasts impairs lysosomal function and thereby inhibits degradation during autophagy (Huang et al., 2019).

Rapamycin is an inhibitor of mTOR, a serine/threonine protein kinase of the phosphatidylinositol 3-kinase-related kinase family. mTOR is sensitive to a variety of environmental and endocrine stimuli and has important regulatory functions in cell proliferation, metabolism, and the aging process (Arriola Apelo and Lamming, 2016). Rapamycin has previously been successfully used in mouse models of age-related diseases, including cancer and Alzheimer's disease, and it has been demonstrated to improve age-related cognitive decline and even rejuvenate aged hearts in mice (Wilkinson et al., 2012; Lin et al., 2017). A previous studies has suggested that the “anti-aging” effects of rapamycin are achieved by a reduction in reactive oxygen species (ROS) (Qin et al., 2018), however, the exact role and potential protective effects of rapamycin in UVA-induced photoaging of skin fibroblasts, as well as the role of HSP27 in protecting from photoaging, remain unclear.

Previously, we had used a UVB-induced photodamage model in HaCat cells which displayed a range of senescence phenotypes, including altered cell morphology, positive senescence-associated beta galactosidase (SA-β-gal) staining, and cell cycle arrest (Liu et al., 2019a). Here, to reveal the mechanisms underlying dermal photoaging induced by UVA, we investigated the role of rapamycin and HSP27 in a UVA irradiation human dermal fibroblasts (HDFs) photoaging model, focusing on cellular effects including UVA-irradiated cell autophagy.

## Materials and Methods

### Cells

Healthy skin tissues were obtained from patients who underwent skin biopsies at our hospital from December 2018 to July 2020. All procedures were approved by the Ethics Committee of The First Affiliated Hospital of Chongqing Medical University, and all patients provided written informed consent. Healthy skin tissues were cut into fragments under aseptic conditions, and the epidermis and dermis were separated to obtain primary human dermal fibroblasts (HDFs). Tissues were digested with type II collagenase (Sigma-Aldrich, USA) and cultured in Dulbecco's modified Eagle medium (DMEM, Gibco, USA) with 10% fetal bovine serum (FBS, Absin, China) at 37°C under 5% CO_2_. This study was conducted using two to four generations of fibroblasts.

### Ultraviolet a (UVA) Irradiation Cell Model

For all experiments, HDFs with high cell viability and rapid proliferation were selected. In the UVA irradiation group, fibroblasts were irradiated with 5 J/cm^2^ once per day for 8 days using a UVA lamp (TL10RS, Philips, Netherlands) with an emission spectrum between 320 and 400 nm. UVA irradiation was measured by a UVA 365 radiometer (Photoelectric Instrument Factory of Beijing Normal University, China). Control cells were exposed to the same conditions but not UVA-irradiated.

### Evaluation of Cellular Viability

The viability of HDFs following exposure to different doses of UVA and different concentrations of rapamycin were assessed using the CCK-8 assay (MCE, USA). Cells were incubated with MTT solution for 1 h, and absorbance was measured at 450 nm using Multiskan Spectrum(ThermoFisher Scientific, USA).

### RNA Isolation and Real Time PCR (RT-PCR)

Total RNA was extracted using the RNAiso Plus kit (TaKaRa, Japan) and converted to cDNA using the RT Master Mix for qPCR (MCE, USA). The SYBR Green qPCR Master Mix (MCE, USA) was used for RT-PCR. For each reaction, a total of 35 cycles were carried out. Primer sequences were as follows:

**Table:** 

**Name**	**Forward primer (5'->3')**	**Reverse primer (5'->3')**
Collagen 1	AAGGTGTTGTGCGATGACG	GGTTTCTTGGTCGGTGGGT
MMP-1	GGCTGAAAGTGACTGGGAAAC	CTTGGCAAATCTGGCGTG
MMP-3	CAATCCTACTGTTGCTGTGCG	CAAGGTTCATGCTGGTGTCC
MMP-9	TTTGACAGCGACAAGAAGTGG	TCAGGGCGAGGACCATAGAG
p62	TGAGTCCCTCTCCCAGATGCT	GGGGGATGCTTTGAATACTGG
HSP27	CCCACCCAAGTTTCCTCCT	GGCAGTCTCATCGGATTTTG
LC3 B	AAGGCGCTTACAGCTCAATG	ACACTGACAATTTCATCCCGA
ACTB	AGAAAATCTGGCACCACACCT	GATAGCACAGCCTGGATAGCA

### Western Blotting

RIPA lysis buffer (Beyotime Biotechnology, China), including Protease Inhibitor Cocktail (MCE, USA), was used to extract proteins. The total protein concentration in each sample was assessed using the BCA assay (Beyotime Biotechnology, China). Proteins were separated on 10 and 12% polyacrylamide gels using SDS-PAGE and transferred to nitrocellulose membranes. Sequentially, the membranes were blocked in 7% skimmed milk for 1 h at room temperature and incubated with primary antibodies and secondary antibodies. Finally, protein bands were visualized using the SuperSignal™ West Femto Maximum Sensitivity Substrate kit (ThermoFisher Scientific, USA). Protein bands were quantified by densitometry and normalized to β-actin. The antibodies used in this study: HSP27(Abcam, UK), Phosphorylated-HSP27, beta-Actin, p53, COL1A1(Cell Signaling Technology, USA), p62, LC3 B, ATG5 (Bimake, USA).

### siRNA Transfection

siRNAs were obtained from GenePharma (Shanghai, China). The siRNA sequences used to inhibit HSP27 expression were 5'-GGACGAGCAUGGCUACAUCTT-3' (sense strand) and 5'- GAUGUAGCCAUGCUCGUCCTT-3' (antisense strand). HDFs with good cell viability and rapid proliferation were selected for transfection. Fibroblasts were inoculated in 6-well plates and pre-cultured for 24 h, and experiments were performed after the cells were completely walled. The complete medium was then replaced by serum-free medium and siRNA and HiPerFect Transfection Reagent (Qiagen, Germany) were added. Transfection continued for 6 h before replacing serum-free medium with complete medium for subsequent experiments.

### Senescence-Associated Beta-Galactosidase (SA-β-gal) Staining

Cells were stained using the Senescence β-Galactosidase Staining Kit (Beyotime Biotechnology, China) according to the manufacturer's instructions. At room temperature, the medium was discarded and cells were washed once with PBS and fixed with 4% polymethanol for 15 min. Cells were again washed three times with PBS and incubated with the staining solution overnight at 37°C. Samples were observed and counted under a reverse-phase microscope (200x) (Olympus, Japan), and five randomly collected images were used for quantification of senescent cells.

### Histological Analysis

We selected pathological tissue specimens for immunohistochemical labeling and Masson staining from five patients with a clinical diagnosis of chronic actinic dermatitis and six patients have normal skin with cosmetic surgery. All the patients signed informed consent. Clinically removed skin specimen were fixed with 4% paraformaldehyde and then paraffin-embedded for sectioning. Each specimen was subjected to hematoxylin-eosin staining, Masson-trichrome staining, and immunohistochemical labeling. Under the microscope, five randomly selected fields of view were used to collect images for subsequent analysis.

### Flow Cytometry to Detect Apoptosis

Twenty-four hours after the last UVA irradiation, cells grown in the 6-well plates as well as the supernatant were collected, labeled with Annexin V-fluorescein isothiocyanate and propidium iodide, and apoptotic cells were detected by flow cytometry (Beckman Coulter, UK). The number of apoptotic cells was analyzed using the Flowjo_v10 software.

### Statistical Analysis

All experiments were repeated at least three times. Statistical analyses were performed using Graphpad Prism 8 (GraphPad Software Inc., USA) and SPSS v22.0 (IBM Corporation, USA), and data are presented as mean ± SD. Between-group comparisons were performed using ANOVA or student's *t*-test for statistical analysis. *p* < 0.05 was considered statistically significant.

## Results

### Optimization of UVA and Rapamycin Dosage

**Figure 1:**
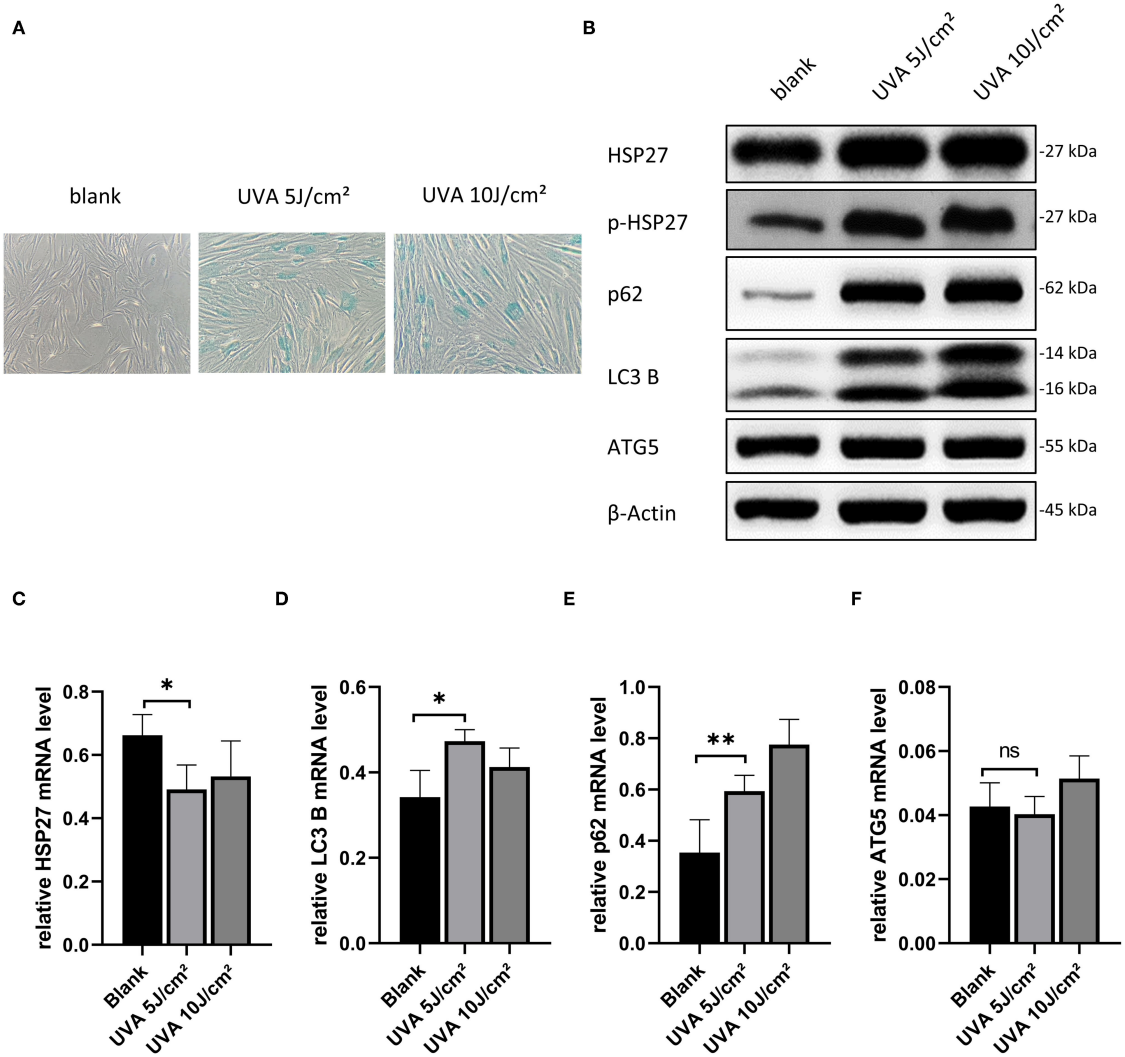
Model construction of UVA-induced photoaging of HDFs and selection of rapamycin concentration. **(A)** SA-β-gal stained images of HDFs after 5 and 10 J/cm^2^ UVA irradiation, original magnification (200x). **(B)** Western blots of HSP27, p-HSP27, p62, LC3 B, and ATG 5 proteins 24 h after the last UVA irradiation. Expression levels were quantified using β-actin as a reference. **(C–F)** RT-PCR for mRNA expression levels of HSP27, p62, LC3 B, and ATG 5. These results are expressed as the mean ± SD of at least three independent experiments (*p < *0.05*, **p < *0.01*, ***p < *0.005*).

Based on previously published work, we initially assessed the effect of 5, 10, and 20 J/cm^2^ for our UVA-induced photoaging model in primary human dermal fibroblasts (Nakyai et al., 2017; Xu et al., 2018; Souza et al., 2020). As we observed a high level of cell death at 20 J/cm^2^, we proceeded using 5 and 10 J/cm^2^ for HDF treatment in subsequent experiments. As shown in Figure 1A, there were significantly more senescent (SA-β-gal positive) HDFs after UVA irritation, but there was no difference between the 5 and 10 J/cm^2^ UVA irritation groups. Moreover, at the protein level, there was a significant increase in the expression of p-HSP27 and p62, and a decrease in LC3 II/LC3 I in both UVA irritation groups as compared to controls (Figure 1B). The same results were also reflected in the mRNA level (Figures 1C–E). Levels of ATG5 correspond to the early stages of autophagosome formation. These were not significantly altered after UVA irritation (Figures 1B,F), neither at mRNA nor protein level. As our results showed that a relatively low dose of UVA is sufficient to induce photoaging, we chose UVA irradiation of 5 J/cm^2^ for our model.

**Figure 2:**
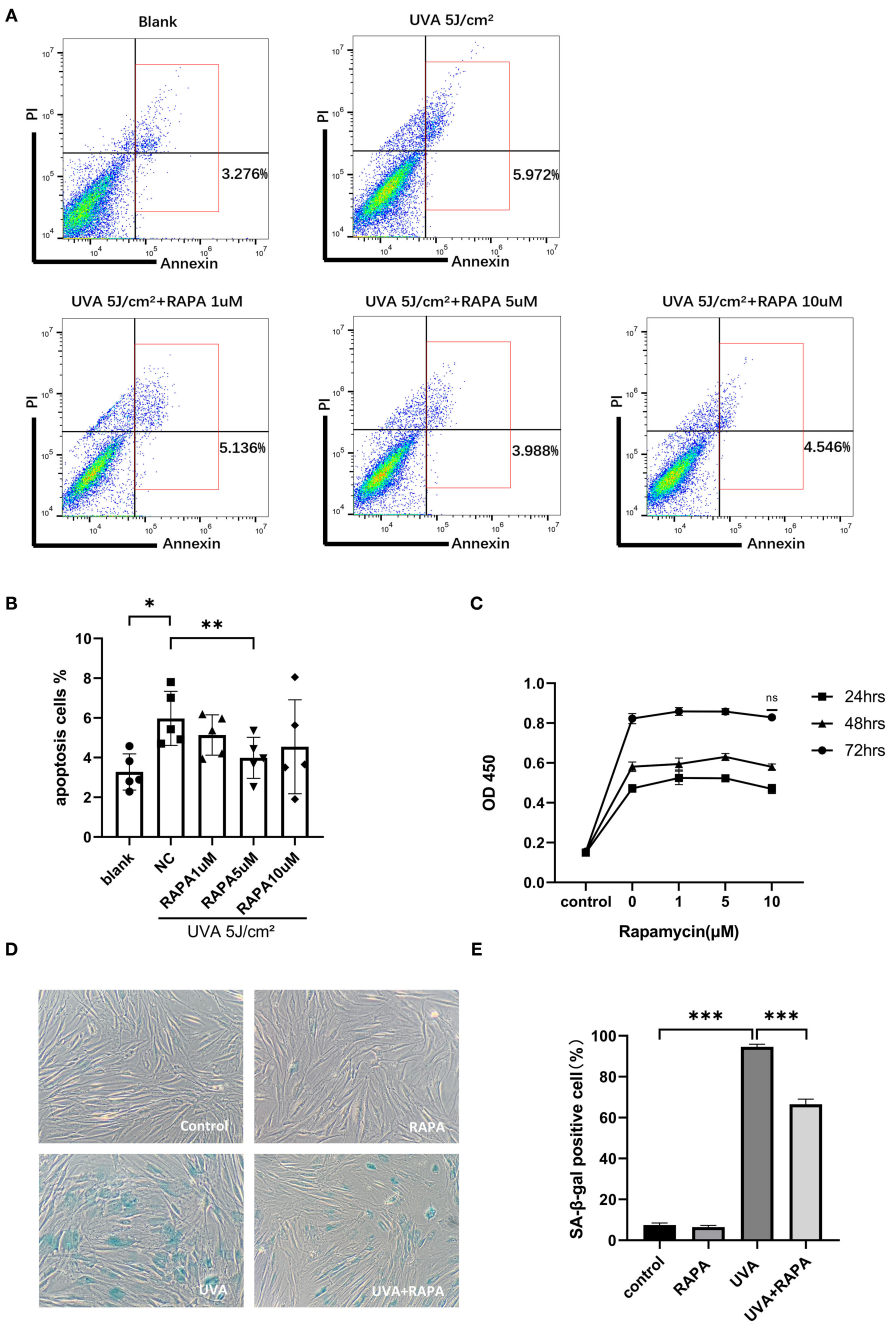
Selection of rapamycin concentration and SA-β-gal staining of HDFs. **(A,B)** Flow cytometry showed that UVA significantly increased apoptosis in HDFs and 5 μM rapamycin was effective in reducing UVA-induced apoptosis of HDFs. **(C)** The proliferative activity of HDFs after 24, 48, and 72 h incubation with rapamycin (0–10 μM) was analyzed using the CCK-8 colorimetric assay, and results are expressed as OD ± SD. This revealed no statistical difference compared with the control group. **(D)** There were significantly more SA-β-gal(+) cells in the UVA group and fewer and lighter staining in the UVA+rapamycin group than in the UVA group. Few SA-β-gal(+) cells are seen in the control and rapamycin groups. **(E)** SA-β-gal positivity was calculated based on 400 cells counted per well. Results are expressed as mean ± SD of at least three independent experiments (*p < *0.05*, **p < *0.01*, ***p < *0.005*).

To determine the optimal concentration of rapamycin for subsequent experiments, we evaluated the cytotoxic and anti-apoptotic effects of rapamycin on primary HDFs in the concentration range of 0–10 μM. Our results showed that there is no cytotoxic effect of rapamycin at 0–10 μM (Figure 2C), while 5 μM of rapamycin showed an anti-apoptotic efficacy in UVA-treated cells (Figures 2A,B).

### Rapamycin Reduces UVA-Induced Cellular Senescence

HDFs in the UVA irradiation group showed a large degree of senescence in the UVA irritation group compared to the control group, but this increase in senescence could be reversed by rapamycin treatment (Figures 2D,E).

### Rapamycin Promotes Autophagy in UVA-Irradiated HDFs

**Figure 3:**
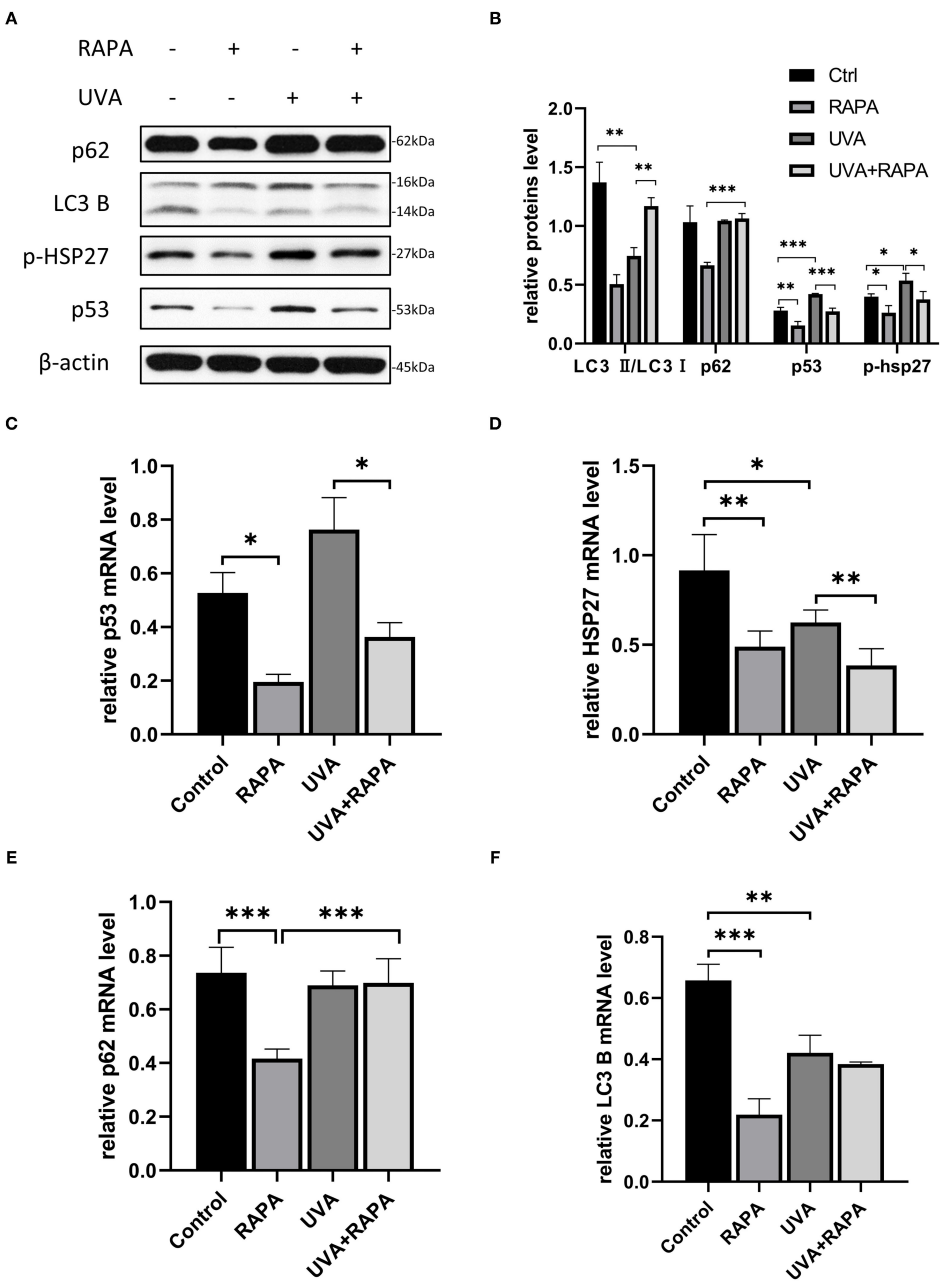
5μM Rapamycin treatment of UVA-induced photoaged HDFs promoted increased autophagy, reduced the expression of p-HSP27 and p53, and decreased the stress levels of cells. **(A,B)** Western blots of p62, LC3 B, p-HSP27, and p53 expression after 24 h of rapamycin treatment. Proteins were quantified using β-actin as reference and expressed as a fold change in protein levels compared to control cells. Results are expressed as mean ± SD of at least three experiments (*p < *0.05*, **p < *0.01*, ***p < *0.005*). **(C–F)** RT-PCR for mRNA expression levels of p62, LC3 B, p-HSP27 and p53. Results are expressed as mean ± SD of at least three assays (*p < *0.05*, **p < *0.01*, ***p < *0.005*).

Light chain protein 3 (LC3) is a key molecule in the autophagy pathway. In the presence of autophagic activation, LC3I converts to LC3II and binds to the membrane of the autophagosome, thus the LC3II/LC3I ratio is widely used to assess autophagy levels (Li et al., 2011). HDFs irradiated with UVA showed a significant decrease in LC3II/LC3I expression compared to the control group, while there was no significant change in p62, which is a substrate for the autophagic degradation phase (Lamark et al., 2017), indicating that UVA causes the cellular autophagic flow to stagnate in the degradation phase. However, the expression of both p53 and p-HSP27 was significantly increased, indicating that HDFs exhibited elevated stress levels in response to UVA. Both with and without UVA irradiation, the autophagic flux was significantly increased in rapamycin-treated HDFs, while the expression of both p53 and p-HSP27 was significantly decreased, suggesting that rapamycin may be able to counter UVA-induced increases in cellular stress levels. However, surprisingly, p62 levels following UVA irradiation remained unchanged even after activation of the autophagy pathway with rapamycin, further confirming that UVA irradiation induces an impairment of degradation during autophagy (Figure 3).

### Effect of Rapamycin on HDFs Following siRNA Knockdown of HSP27

**Figure 4:**
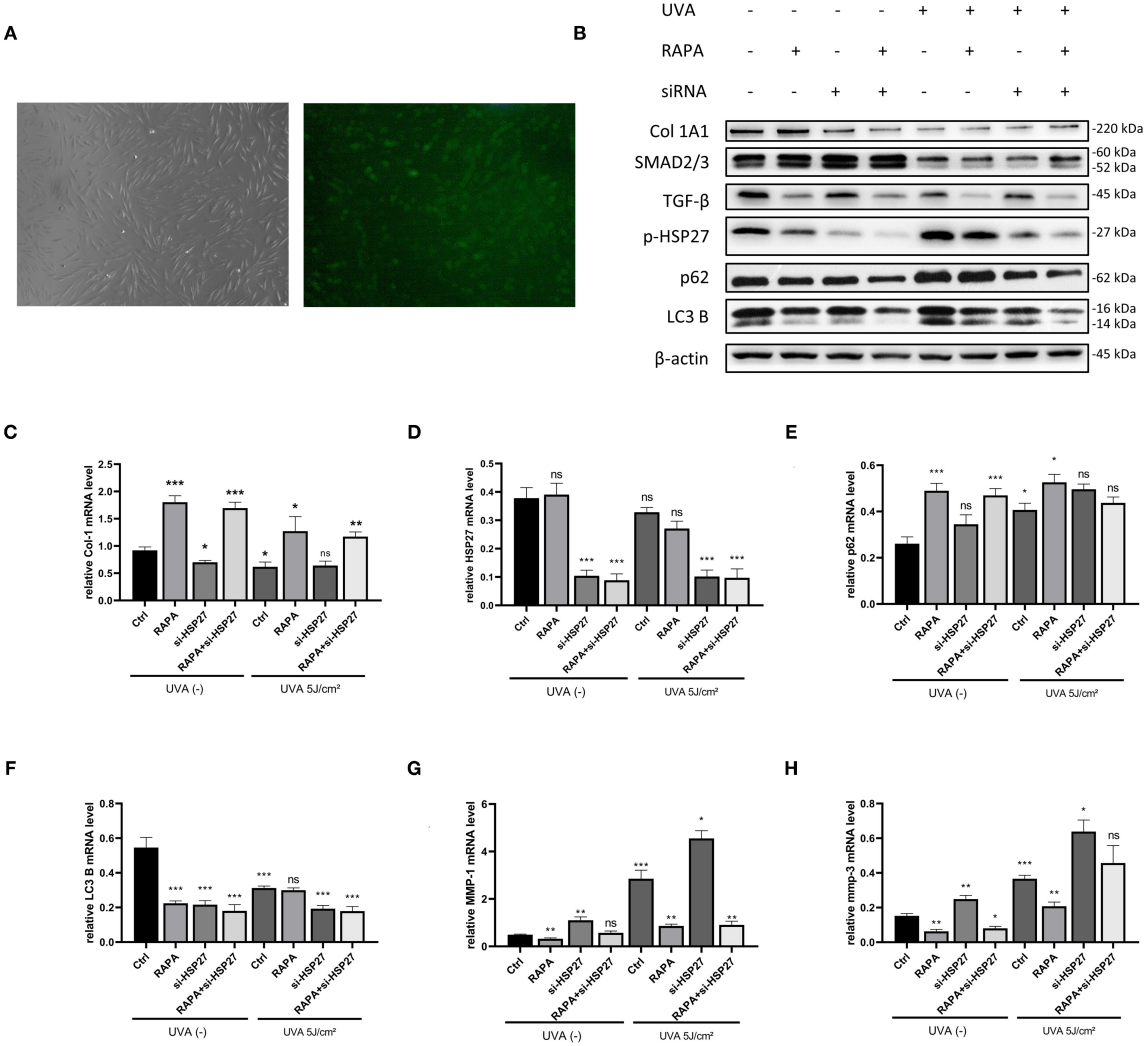
5μM Rapamycin treatment of UVA-induced photoaged si-HSP27 HDFs reduced the collagen reduction and MMP-1 and 3 expression following UVA irradiation and knockdown of HSP27. **(A)** Fluorescence microscopy of FAM-labeled HSP27 siRNA-transfected fibroblasts at original magnification (40x), transfection efficiency around 80%. **(B)** Western blot of Collagen I, TGF-β,SMAD2/3,p-HSP27, p62, and LC3 B expression of knockdown HSP27 HDFs after 24 h of rapamycin treatment. Proteins were quantified using β-actin as reference and expressed as a fold change in protein levels compared to control cells. Results are expressed as mean ± SD of at least three assays (*p < *0.05*, **p < *0.01*, ***p < *0.005*). **(C–H)** RT-PCR was performed to detect the mRNA expression levels of Collagen I, p-HSP27, p62, and LC3 B. The mRNA expression levels of Collagen I, p-HSP27, p62, and LC3 B were determined by RT-PCR. Results are expressed as mean ± SD of at least three assays (*p < *0.05*, **p < *0.01*, ***p < *0.005*).

To investigate the effect of HSP27 on autophagy in UVA-induced photoaging HDFs, as well as the role of rapamycin in HDFs, we constructed HSP27 knockdown HDFs by using HSP27 siRNA (Figures 4A,D).We subsequently detected the expression of autophagy-associated p62 and LC3B in the control and si-HSP27 group by western blotting, and found that HSP27 knockdown suppressed the expression LC3B expression regardless of UVA irradiation, while it increased the expression of p62 following UVA exposure (Figures 4B,E,F).

It has been reported that UVA can significantly decrease the expression of type I collagen and increase the expression of MMP-1, 2, 3, and 9 in photoaged cells (Pittayapruek et al., 2016), and we have previously observed similar results in our models. Interestingly, regardless of UVA exposure, we found a significant increase in the expression of MMP-1 and 3, as well as a significant decrease in type I collagen mRNA levels following knockdown of HSP27 in HDFs. However, this effect was alleviated in the rapamycin-treated group. Therefore, we hypothesize that HSP27 may be involved in the synthesis of MMPs and collagen degradation, and rapamycin could alleviate alterations induced by HSP27 deficiencies or dysfunction (Figures 4B,C,G,H).

### Expression of Type I Collagen, HSP27 and Autophagy-Related Proteins in Clinical Tissues

**Figure 5:**
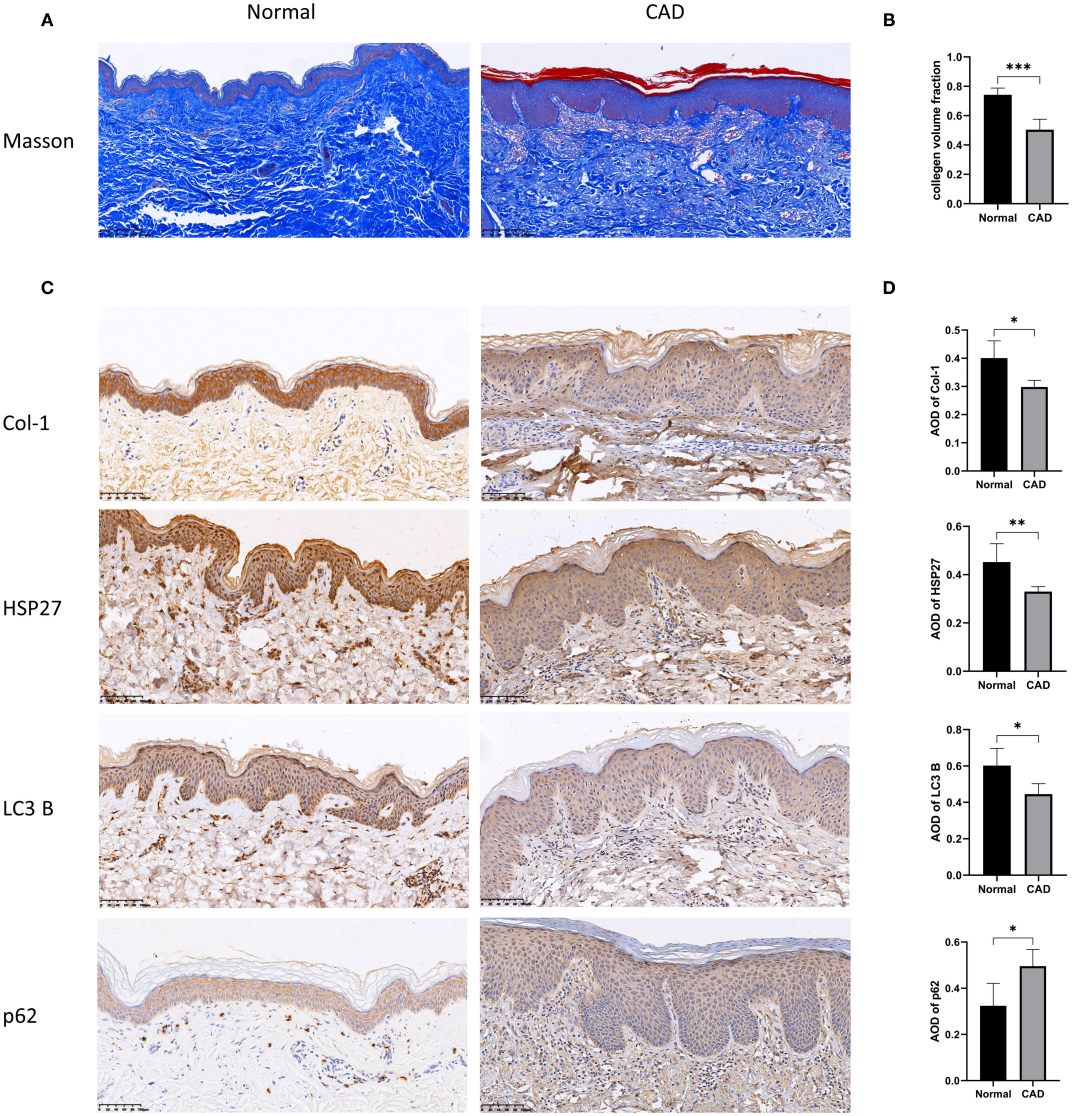
Histopathological results of the skin of normal and photoaged groups using Masson trichrome staining and immunohistochemical labeling. **(A,B)** Histopathological results of Masson staining of skin from normal and photoaged groups. The collagen volume fraction was calculated, which is expressed as mean ± SD of at least three measurements (*p < *0.05*, **p < *0.01*, ***p < *0.005*). **(C,D)** Immunohistochemical markers for Collagen I, HSP27, p62 and LC3 B and quantification of Average Optical Density (AOD) were performed on normal and photoaged skin, and the results are expressed as mean ± SD of at least three measurements (*p < *0.05*, **p < *0.01*, ***p < *0.005*).

To further investigate the relationship between HSP27, autophagy and type I collagen, we selected tissues from normal skin biopsies and from patients with a diagnosis of chronic actinic dermatitis (CAD) for Masson staining and immunohistochemical labeling of HSP27, LC3 B, p62, and Collagen I. Compared to normal patients, skin tissue from patients with photoaged dermatitis expressed lower levels of HSP27, LC3 B, and Collagen I, while p62 expression showed diffuse flaky positive foci. Masson staining revealed that the dermal collagen in CAD patients was partially pale, disorganized and loose, and the collagen fibers were significantly reduced. These results suggested that photoaged skin exhibits lower levels of autophagy and collagen fibers than normal skin, which was consistent with our *in vitro* experiments (Figure 5).

## Discussion

Dermal fibroblasts are the major cellular component of the dermis, producing collagen, fibronectin, glycosaminoglycans and other extracellular matrix components which support the dermal structure and help to maintain skin elasticity and moisture (Thulabandu et al., 2018). UVA is a potent form of UV radiation that can damage dermal fibroblasts. UVA exposure leads to fibroblast cell cycle arrest, apoptosis, dysfunction of secretion and synthesis and skin aging (Wlaschek et al., 2001). The skin of the head and face is particularly susceptible to photoaging due to its continuous and long-term exposure to the environment. In order to avoid intrinsic photoaging, we selected skin tissues from non-exposed regions for our primary fibroblast culture in this study. There is currently no “gold standard” for *in vitro* modeling of UVA-induced fibroblast photoaging. Based on the literature and previous studies conducted in our group, we initially tested irradiation with 5, 10, and 20 J/cm^2^ UVA on HDFs, respectively, to identify the appropriate irradiation dose. This revealed that, with the exception of 20 J/cm^2^ UVA irradiation which induced significant cell death, both 5 and 10 J/cm^2^ UVA caused significant senescent morphological changes in HDFs as well as positive SA-β-gal staining. As we aimed to model chronic photoaging in this project, we chose the lower dose of 5 J/cm^2^ of UVA as the irradiation dose for our experimental model. Hereby, we provide a new *in vitro* model of UVA-induced fibroblast photoaging.

HSP27 has previously been considered as an adaptive cellular response protein that helps the organism to survive due to its protective effects against heat-induced cellular damage and its primary function as a molecular chaperone involved in protein folding and a variety of cellular processes (Singh et al., 2017). Additionally, the phosphorylated form of HSP27 can interact with denatured and aggregated proteins to protect cells against severe damage from stress (Kampinga et al., 1994; Trott et al., 2009). In previous studies, we have demonstrated that HSP27 is significantly elevated in photoaged rat skin and HaCaT cells and exerts protective effects against photoaging by modulating anti-apoptotic pathways (Liu et al., 2019a,b). In our UVA photoaging model, we found no significant increase in the expression of HSP27, while phosphorylated HSP27 increased significantly. This suggested that p-HSP27 may be more important for protection from photoaging in HDFs.

Rapamycin is a classical activator of autophagy which acts by inhibiting the activity of mTOR (Arriola Apelo and Lamming, 2016). Autophagy is activated following UVA-induced genotoxic stress and elevated ROS levels (Galati et al., 2019). Rapamycin has been reported to reduce UVB-induced ROS accumulation and promote the antioxidant capacities of cells (Qin et al., 2018). In our study, p-HSP27 was significantly decreased after rapamycin treatment, which also seems to suggest that rapamycin reduced the level of UVA-induced oxidative stress on cells. The tumor suppressor p53 is involved in a variety of cellular processes such as apoptosis, necrosis, aging and autophagy, and is also a major constituted of the DNA damage response pathway (Mrakovcic and Frohlich, 2018). In our UVA-induced photoaging model of HDFs, a significant elevation of p53 was accompanied by activation of the autophagy pathway, and treatment with rapamycin inhibited the sustained increase in p53 expression despite elevated levels of cellular autophagy, preventing programmed cell death due to over-activation of autophagy. Taken together, these results indicate a potential of rapamycin to protect against oxidative and genotoxic stress, and despite the many well-documented adverse effects of rapamycin as an immunosuppressant, our experimental results suggest it may be suitable as a anti-photoaging agent.

Repeated exposure to sunlight causes skin photoaging, which is characterized by coarse, dry skin texture, pigmentation disorders, increased wrinkles, and capillary dilatation. The mechanisms underlying skin photoaging have been suggested to involve alterations in cellular composition, a significant reduction in dermal collagen, accumulation of abnormal elastic fibers, and degradation of extracellular matrix components (Amano, 2016). It has been shown that UVA-induced photoaging increases the expression of MMPs and inhibits the synthesis of type I collagen (Varani et al., 2001), which we were able to confirm in this study as the expression of MMP-1 and MMP-3 was significantly increased, while the expression of type I collagen was significantly decreased in UVA-irradiated HDFs. Interestingly, when HSP27 was knocked down, a further exacerbation of the UVA-induced increase in MMPs expression and degradation of type I collagen was observed. This may suggest that HSP27 is involved in the regulation of MMPs and collagen, and exerts its effects in anti-photoaging, at least in part, via these cellular components. After rapamycin treatment, the effects of UVA irradiation and knocked down HSP27 were abolished: the expression of MMPs was decreased and an increased synthesis of type I collagen was observed. These effects of rapamycin might be achieved via activation of the classical TGF-β/Smad signaling pathway and the transcriptional activation of MAPK/AP-1 (Wu et al., 2018). However, in our results, rapamycin was not shown to increase the expression of TGF-β, but significantly decreased it. Rapamycin has been reported to directly activate the TGF-β receptor (Miyakawa et al., 2018), thus activating the TGF-β/SMAD pathway and increasing the expression of type I collagen.

Despite promising findings in this study, we have not yet been able to determine in what way rapamycin regulates HSP27 to exert its anti-photoaging effects and can thus far only speculate about the potential pathways by which this regulation may occur. In future studies, we will continue to explore the mechanisms of the regulation of autophagy and anti-photoaging by HSP27 in depth.

## Data Availability Statement

The original contributions generated for the study are included in the article/supplementary material, further inquiries can be directed to the corresponding author/s.

## Ethics Statement

The studies involving human participants were reviewed and approved by The first affiliated hospital of Chongqing Medical University of ethics committee. The patients/participants provided their written informed consent to participate in this study.

## Author Contributions

G-LB and PW performed all experiments, wrote the manuscript, and interpreted the data. XH and A-JC provided technical support for this study. Z-YW, DC, CL, Y-YL, and R-LL reviewed the literature. G-LB and PW contributed equally to this work. All authors examined the manuscript and approved the conclusions.

## Conflict of Interest

The authors declare that the research was conducted in the absence of any commercial or financial relationships that could be construed as a potential conflict of interest.
